# Supplementary material for: Description of Ultra-Processed Food Intake in a Swiss Population-Based Sample of Adults Aged 18 to 75 Years
Source: Nutrients. 2022 Oct 25;14(21):4486. doi: 10.3390/nu14214486 (PMC9659134; doi:10.3390/nu14214486)
Supplement: Supplementary file 1 [file nutrients-14-04486-s001.zip › nutrients-1966600-supplementary.pdf]

nutrients

MDPI

Supplementary Materials

Description of ultra-processed food intake in a Swiss population-based sample of adults aged 18 to 75 years

Valeria A. Bertoni Maluf et al.

Table S1. Eighteen foods groups and examples of foods from each food group

N°	Food groups	Examples
1	Cereal products, legumes & potatoes	Tubers and tuber products; Pasta and pasta products; Flours and starches; Dough; Rice; Bread and bread products (fresh or **pre-packaged bread**); Legumes; Crackers; Cereal flakes (natural).
2	Fruit & vegetables	All types of fruits.<br>Except: fruit juices, fruit jams or candied fruits.<br>All types of vegetables (green salads/leaves, sprouts, mushrooms and seaweeds, sweet corn, snow peas, pumpkin, fresh green beans, onions).<br>Except: avocadoes, olives, herbs, vegetable juices or sauces (e.g. Ketchup, pesto sauce).
3	Dairy products	Milk and milk products; Yogurt (plain or **flavoured yogurt**) and yogurt products; Cheese.
4	Meat, fish & eggs	Unprocessed meat; Fish and seafood; Eggs.<br>**Processed meat; Processed fish products**.
5	Added fats	Vegetable oils; Butter; **Margarine**; Cream.
6	Nuts & seeds	Nuts and seeds; Avocadoes; Olives.
7	Industrial dishes	Vegetarian products and meals (Falafel); Rolls and croissants with spreads or fillings, savoury pies.
8	Soups & broth	All soups and broth (homemade and **industrial)**.
9	Juices & soft drinks	**Soft drinks** (sugary and sugar-free); **Sports drinks**, **energy drinks**, **fizzy drinks**, iced tea, diluted syrup; Fruit and vegetable juices.
10	Other non-alcoholic beverages	Coffee, tea, fruit and herbal tea; Water.
11	Alcoholic beverages & substitutes	Alcohol beverages (beer, wine, spirits, cocktails); **Alcoholic drink substitutes** (alcohol-free beers, punch and cider, all without alcohol).
12	Sugar, honey, jam, sweet sauces & syrups	Sugar; Honey; Marmalade; Jam; Sweet sauces and syrups.
13	Ice-creams & milk-based desserts	Milk-based and water-based ice creams and milk-based desserts (crème brûlée, chocolate mousse).
14	Breakfast cereals	**Breakfast cereals** (processed and branded breakfast cereals).
15	Confectionary, cakes & biscuits	Chocolate and confectionery; Cakes, sweet pies and pastries; Biscuits (homemade or **pre-packaged biscuits**).
16	Salty snacks	**Ready-to-eat salty snacks** (All types of crisps and flips, salty crackers, salty popcorn, etc.).
17	Seasoning, spices, yeast & herbs	All sauces (homemade and **industrial sauces**); Spices, herbs and flavourings; Vinegar.
18	Other foods	Tofu and **meat substitutes**; Yeast; Added **artificial sweeteners**; Cream substitutes.

*Ultra-processed food examples are in **bold**.

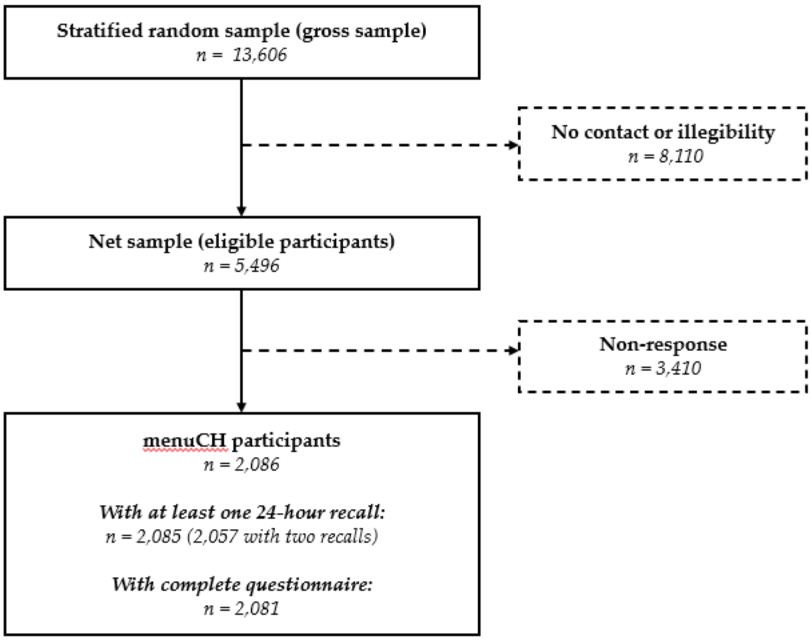

Figure S1. Flowchart showing causes of participants exclusion from analyses (menuCH, Swiss National Nutrition Survey, 2014-15).

Table S2. Distribution of daily total intake (grams/day) and intake from UPFs (grams/day) by food group in decreasing order (N=2,085, **bold** = 3 largest numbers, *italic* = 3 smallest number, by column).

**Food Groups**	**Total intake** (g/day)	**Contribution to total diet weight** (%TDW)	**UPFs intake** (g/day)	**UPFs intake from total intake** (%) [1]	**UPF intake from total diet weight** (%TDW) [2]
	Mean (SD)	*Mean (SD)*	*Mean (SD)*	*Mean (SD)*	*Mean (SD)*
Juices & soft drinks	277 (408)	8.0 (10.8)	**210 (387)**	58.9 (43.5)	**26.0 (30.6)**
Confectionary, cakes & biscuits	51 (57)	1.5 (1.8)	**50 (56)**	**99.6 (50.4)**	**15.9 (19.0)**
Dairy products	219 (193)	6.5 (5.5)	**48 (87)**	18.7 (28.2)	**11.1 (18.9)**
Meat, fish & eggs	132 (96)	3.9 (2.8)	38 (49)	31.2 (32.2)	10.9 (15.5)
Cereal products, legumes & potatoes	**281 (147)**	**8.4 (4.4)**	25 (37)	10.5 15.9)	7.8 (13.1)
Seasoning, spices, yeast & herbs	49 (49)	1.5 (1.4)	15 (30)	28.8 35.7)	4.5 (9.6)
Added fats	32 (32)	1.0 (1.0)	11 (24)	23.2 (31.3)	3.6 (8.6)
Alcoholic beverages substitutes	188 (311)	5.3 (8.1)	19 (72)	13.2 (29.5)	3.5 (11.1)
Other foods	1 (53)	0.4 (1.5)	13 (52)	**96.0 (19.2)**	3.5 (12.5)
Soups & broth	48 (101)	1.4 (3.0)	13 (50)	41.2 (47.9)	3.0 (10.9)
Ice-creams & milk-based desserts	14 (35)	0.4 (1.0)	9 (24)	74.5 (41.9)	2.6 (7.9)
Sugar, honey, jam, sweet sauces & syrups	21 (26)	0.6 (0.8)	7 (17)	27.5 (38.7)	2.2 (6.5)
Other non-alcoholic beverages	**1751 (820)**	**49.8 (15.4)**	11 (70)	*0.6 (3.5)*	1.8 (9.2)
Salty snacks	*5 (16)*	*0. 1 (0.5)*	5 (16)	**100 (0.0)**	1.5 (5.3)
Breakfast cereals	*7 (18)*	*0.2 (0.5)*	5 (15)	65.8 (44.9)	1.4 (5.2)
Industrial dishes	*5 (24)*	*0.1 (0.7)*	*2 (17)*	40.4 (48.4)	*0.5 (3.6)*
Nuts & seeds	10 (21)	0.3 (0.6)	*0 (0)*	*0.0 (0.0)*	*0.0 (0.0)*
Fruit & vegetables	**341 (226)**	**10.1 (6.3)**	*0 (0)*	*0.0 (0.0)*	*0.0 (0.0)*
Total	3,443 (981)	100.0	481 (463)	-	100.0

[1] Among consumers only (N varies according to food groups, e.g., N=2,074 for Cereal products, legumes & potatoes to N=155 for Industrial recipes & fast-food dishes). [2] Seven people did not consume any UPFs (N_{total}=2,078).

Table S3. Nutrient profile of the overall diet and of ultra-processed products (N=2,085) and missing values from the Food Composition Database, by nutrient

Nutrients	Total daily intake [1]	Total daily intake from UPFs	Relative contribution of UPFs to total intake (%) [2]	Missing values for all foods [3]	Missing values for UPFs
	Medians (P25-P75)	Medians (P25-P75)	Medians (P25-P75)	N (%)	N (%)
**Proteins (g)**	**76.0 (60.3-97.8)**	**15.7 (9.0-25.5)**	**21.0 (12.6-32.2)**	**0**	**0**
**Total carbohydrates (g)**	**211.9 (164.6-273.3)**	**61.7 (36.0-100.4)**	**30.7 (19.6-43.4)**	**0**	**0**
**Dietary fibre (g)**	**18.7 (14.0-24.4)**	**2.7 (1.4-4.8)**	**15.2 (8.1-25.9)**	**2,822 (2.3)**	**1,107 (4.8)**
**Sugars (g)**	**94.5 (67.5-128.3)**	**34.0 (18.1-60.1)**	**39.3 (24.0-57.2)**	**3,971 (3.3)**	**989 (4.3)**
**Total fats (g)**	**83.6 (64.0-107.4)**	**25.4 (14.9-40.5)**	**31.8 (20.6-44.6)**	**0**	**0**
PUFAs (g)	7.4 (5.1-10.7)	1.3 (0.5-2.7)	19.1 (7.4-35.2)	25,121 (20.8)	10,002 (43.5)
MUFAs (g)	21.0 (14.4-28.9)	4.3 (1.8-8.4)	22.4 (9.5-40.2)	25,121 (20.8)	10,002 (43.5)
**SFAs (g)**	**31.0 (22.6-41.6)**	**9.8 (5.4-16.3)**	**32.8 (21.6-46.8)**	**5,027 (4.2)**	**1,192 (5.2)**
Cholesterol (mg)	210.0 (135.0-320.1)	26.9 (8.3-59.2)	12.6 (4.4-28.5)	21,772 (18.0)	10,223 (44.4)
Calcium (mg)	644.9 (455.8-896.3)	42.9 (16.5-100.0)	7.1 (2.6-14.8)	24,554 (20.3)	10,810 (47.0)
Chloride (mg)	2674.6 (1856.5-3767.5)	458.7 (148.6-1172.9)	19.4 (6.9-37.8)	27,795 (23.0)	11,900 (51.7)
Iodine (mg)	55.4 (38.6-80.1)	4.0 (1.5-8.0)	7.2 (2.7-14.8)	26,707 (22.1)	11,854 (51.5)
Iron (mg)	6.7 (4.8-8.9)	1.0 (0.4-1.9)	16.2 (7.3-28.4)	25,432 (21.0)	10,945 (47.6)
Magnesium (mg)	204.5 (160.5-256.0)	23.2 (10.8-41.4)	11.4 (5.4-20.6)	24,658 (20.4)	10,882 (47.3)
Phosphorus (mg)	855.6 (649.6-1104.1)	115.8 (49.5-204.0)	13.3 (6.4-22.9)	25,806 (21.3)	11,237 (48.9)
Potassium (mg)	2039.7 (1571.1-2577.6)	229.6 (106.6-406.1)	11.7 (5.5-20.3)	24,906 (20.6)	11,080 (48.2)
**Sodium (mg)**	**2771.0 (2079.4-3653.9)**	**738.2 (365.1-1304.6)**	**27.8 (15.8-42.9)**	**5,317 (4.4)**	**1,801 (4.7)**
Zinc (mg)	7.1 (5.1-9.8)	1.0 (0.4-1.8)	13.6 (6.2-24.6)	25,631 (21.2)	11,084 (48.2)
Beta-carotene (µg)	2,273.5 (1,033.9-4,353.3)	15.3 (3.1-45.6)	0.7 (0.1-3.1)	31,496 (26.0)	12,534 (54.5)
Cobalamin (µg)	2.9 (1.7-4.8)	0.3 (0.07-0.9)	12.7 (3.2-33.5)	24,564 (20.3)	10,660 (46.3)
Folate (µg)	195.3 (140.3-266.8)	10.1 (3.5-23.4)	5.6 (1.7-13.1)	26,054 (21.5)	11,187 (48.6)
Niacin (mg)	10.2 (7.3-14.8)	1.7 (0.6-3.6)	17.9 (6.7-34.5)	25,595 (21.1)	10,688 (46.5)
Pantothenic acid (mg)	3.4 (2.5-4.4)	0.4 (0.2-0.7)	12.2 (5.3-22.3)	24,931 (20.6)	10,905 (47.4)
Riboflavin (mg)	0.9 (0.7-1.2)	0.1 (0.06-0.3)	15.5 (7.4-27.4)	24,613 (20.3)	10,711 (46.4)
Thiamine (mg)	0.8 (0.6-1.1)	0.1 (0.05-0.3)	19.3 (7.2-37.5)	24,619 (20.3)	10,717 (46.6)
Vitamin A (µg)	570.4 (367.5-837.4)	38.6 (8.9-94.8)	6.8 (1.7-16.9)	25,340 (20.9)	11,095 (48.2)
Vitamin B6 (mg)	1.3 (0.9-1.7)	0.1 (0.1-0.3)	12.2 (4.7-24.6)	24,424 (20.2)	10,534 (45.8)
Vitamin C (mg)	85.5 (51.2-131.5)	5.1 (1.0-14.5)	6.7 (1.1-20.0)	24,734 (20.4)	10,808 (47.0)
Vitamin D (µg)	1.7 (1.0-2.9)	0.2 (0.1-0.6)	14.9 (3.5-36.0)	24,792 (20.5)	10,875 (47.3)
Vitamin E (mg-ATE)	7.6 (5.1-10.8)	0.9 (0.3-2.1)	12.6 (3.7-27.7)	24,459 (20.2)	10,739 (46.7)

[1] Excluding all alcohol drinks. [2] Proportion of the total daily intake brought by UPFs. [3] 121,047 were consumed foods and 23,001 were classified as ultra-processed. **Nutrients with less than 5% of missing data are in bold.**

Table S4. Consumption of UPFs according to time to prepare and cook a hot meal at home[1] (N=2,080 [2]).

Time to prepare and cook a hot meal	Absolute intake of UPFs (Kcal/day) *Mean (SD)*	UPFs consumption (% TEI) *Mean (SD)*	P value for % UPFs [3]
30'or less (N=983)	743 (473)	31.5 (14.4)	<0.001*
More than 30' (N=1,097)	614 (391)	28.9 (14.0)	

[1] Time to prepare and cook a hot meal at home during a usual week was categorized as 30 minutes or less and more than 30 minutes. [2] Five participants did not answer this question. [3] Two-sample Wilcoxon rank-sum (Mann-Whitney) test.
